# Holistic antenatal education class interventions: a systematic review of the prioritisation and involvement of Indigenous Peoples’ of Aotearoa New Zealand, Australia, Canada and the United States over a 10-year period 2008 to 2018

**DOI:** 10.1186/s13690-022-00927-x

**Published:** 2022-07-14

**Authors:** Nikki M. Barrett, Lisette Burrows, Polly Atatoa-Carr, Linda T. Smith, Bridgette Masters-Awatere

**Affiliations:** 1 Te Huataki Waiora School of Health, University of Waikato, Hamilton, New Zealand; 2 National Institute of Demographic and Economic Analysis, University of Waikato, Hamilton, New Zealand; 3 Te Whare Wānanga o Awanuiārangi, Whakatane, New Zealand; 4 School of Psychology, University of Waikato, Hamilton, New Zealand

**Keywords:** Indigenous, Antenatal, Childbirth education, Pregnancy, Maternity, Māori, Aboriginal, First nations

## Abstract

**Background:**

Research into the effectiveness of antenatal education classes is crucial for Indigenous Peoples from Aotearoa New Zealand, Australia, Canada and the United States who experience poorer maternal and infant health outcomes compared to non-Indigenous populations. Our systematic review questions were intended to determine the extent of Indigenous Peoples prioritisation and involvement in antenatal education classes, and to understand the experience of Indigenous Peoples from these countries in antenatal education classes.

**Methods:**

Using a standardised protocol, we systematically searched five electronic databases for primary research papers on antenatal education classes within the four countries noted and identified 17 papers that met the criteria. We undertook a qualitative meta-synthesis using a socio-critical lens.

**Results:**

Systematic review of the academic literature demonstrates that Indigenous Peoples of Aotearoa New Zealand, Australia, Canada and the United States are not prioritised in antenatal education classes with only two of 17 studies identifying Indigenous participants. Within these two studies, Indigenous Peoples were underrepresented. As a result of poor engagement and low participation numbers of Indigenous Peoples in these antenatal education classes, it was not possible to understand the experiences of Indigenous Peoples.

**Conclusion:**

Given that Indigenous Peoples were absent from the majority of studies examined in this review, it is clear little consideration is afforded to the antenatal health needs and aspirations of Indigenous Peoples of Aotearoa New Zealand, Australia, Canada and the United States. To address the stark antenatal health inequities of Indigenous Peoples, targeted Indigenous interventions that consider culture, language, and wider aspects of holistic health must be privileged.

**Trial registration:**

PROSPERO Registration ID: CRD4202017658

## Background

Antenatal care is an opportunity to provide important health-care functions such as medical, physical and educational interventions to expectant mothers [1]. Antenatal education is a core component of antenatal care. Maternal health and wellbeing impacts baby, in utero, after birth, and in future life course health [2–5] and in most developed countries, high quality antenatal education is prioritised to support these crucial life stages.

Childbirth education or antenatal education classes (AEC) aim to prepare prospective parents with skills and knowledge for childbirth and parenthood, in turn supporting improved health outcomes for mother and baby [6–9]. For the last three decades, antenatal education has attracted considerable attention within both practice and research [10–12]. AEC has been a widely accepted practice in many developed countries, particularly within Aotearoa New Zealand (thereafter Aotearoa), Australia, Canada, and the United States (US), though content and delivery style varies both amongst, and within, these countries [13].

In the four countries noted above, maternal and child health inequities between Indigenous Peoples and the dominant population group of the respective country are stark, with Indigenous Peoples experiencing significantly poorer maternal health outcomes [14–17]. The incidence of infant mortality, particularly Sudden Unexpected Death in Infancy (SUDI) which accounts for both explained and unexplained infant deaths, is significantly higher among Indigenous infants [18–20]. These Indigenous populations are also overrepresented in other negative infant health outcomes, such as; greater exposure to cigarette smoke and alcohol while in utero, have lower-birth-weight, higher rates of hospital admission for respiratory illnesses, and lower childhood immunisation rates [21, 22]. These health conditions highlight the need to focus efforts on the health and wellbeing of pregnant mothers during this important period.

Māori are the Indigenous Peoples of Aotearoa. In Canada, there are three Indigenous/Aboriginal groups recognised by the Constitution Act of 1982; First Nations, Inuit, and Métis [23, 24]. In the continental US, the Indigenous peoples are known collectively as Native Americans and, in Alaska, the Indigenous peoples are collectively known as Alaska Native [23]. In Hawaii, a State located approximately 2000 miles off the mainland of the US, Native Hawaiians are the Indigenous population recognised in the Native American Programs Act [25]. Aboriginal and Torres Strait Islander are the Indigenous Peoples of Australia. Each of the four Indigenous population groups have numerous sub tribes/groups, having their own distinct set of languages, histories, and cultural traditions [26]. “Canada, the United States, Australia, and New Zealand consistently place near the top of the United Nations Development Programme’s Human Development Index (HDI) rankings, yet all have minority Indigenous populations with much poorer health and social conditions than non-Indigenous people” [27]. This is a clear breach of the United Nations Declaration on the Rights of Indigenous Peoples (UNDRIP) and reflective of a lack of culturally appropriate and responsive initiatives, contributing to the growing health inequities for Indigenous Peoples [28].

### Framework for health and wellbeing for Indigenous Peoples

In these same countries, programmes and research activities aimed to improve health outcomes have been largely focused on non-Indigenous, rather than Indigenous, understandings of health [23]. Within many Indigenous cultures health and wellbeing goes beyond a biomedical view and is more than the mere absence of illness or disease [29, 30]. Holistic models of health particularly resonate with Indigenous Peoples. Chakanyuka et al. [31] describe holistic health as;

*The vision most First Nations peoples articulate as they reflect upon their future. At the personal level this means each member enjoys health and wellness in body, mind, heart, and spirit. Within the family context, this means mutual support of each other. From a community perspective it means leadership committed to whole health, empowerment, sensitivity to interrelatedness of past, present, and future possibilities, and connected between cultures (p.82).*

Holistic health is a concept echoed by King et al. [23] whereby they expand on how the four life elements, physical, emotional, mental, and spiritual, are privileged amongst Indigenous populations, and that all elements are intricately woven together and interact to support a strong and healthy person. Specific to Māori holistic and whānau-centred (family-centred) approaches have been integral to Māori conceptualisations of health and wellbeing [32]. While each of the Indigenous population groups have their own autonomy and sovereignty over their own health and wellbeing aspirations, they share a collective perspective of health that is intrinsically linked to culture and the environment [33–35].

Siloed health programmes are failing many Indigenous and minority peoples. As King et al. [23] explain, “services and support for health and social programmes are typically fragmented in Indigenous populations…Fragmentation results in the isolation of symptomatic issues—addiction, suicide, fetal alcohol syndrome, poor housing, and unemployment—followed by the design of stand-alone programmes to try to manage each issue separately” (p.83). As opposed to stand-alone programmes, holistic approaches aim to address numerous issues. These holistic approaches to health services align to Indigenous aspirations of health and can support the improvement of Indigenous health.

### Colonisation and its impact on Indigenous birthing knowledge and practices

These four Indigenous population groups share a similar history of colonisation, with the negative impacts still being felt today [36]. Removal of land, the conscious, strategic and forcible loss of identity and culture, and validity of traditional knowledge, are some of the effects of colonisation [37] and each contributes to the inequities of Indigenous health and wellbeing [28].

In Canada, Brown et al. [38] explains that Indigenous Peoples were “…forced into dependency through a system of reserves, compulsory residential schools for children, and a series of policies that prevented the people from pursuing their traditional ways of living and supporting themselves” (p.103). In Australia, the Aboriginal people were “subjected to widespread dispossession, violence, and introduced diseases in the nineteenth century as Europeans took up large areas of country and forced Aboriginal communities onto missions and reserves” ([39] p.82).

In relation to childbirth knowledge, colonisation affected almost all aspects of Māori maternities [40]. Three major factors contributed to the disestablishment of traditional Māori pregnancy, birthing, and parenting, knowledge and practices. First, the introduction of the Western health system, specifically hospital births [41]; Second, the introduction of Western policies and legislations such as the Tohunga Suppression Act [42]; and third, the assimilation of Western family hierarchical structures that ostracised the traditional role of wāhine (women) and tapu (sacredness) of the maternal body [43].

The introduction of hospitals and Western policies have also impacted the Indigenous Peoples of Canada with implementation of Health Canada’s mandated ‘birth evacuation policy’, whereby “all pregnant First Nations and Inuit people (regardless of health risk) living on rural, remote and northern reserves leave their communities near the end of their third trimester and travel to urban hospitals to give birth” ([25] p.173). This policy undermines the voices, experiences, and knowledge of Indigenous women and is an ongoing example of “settler colonialism, white dominance, and national-patriarchy” ([25] p.184).

In contemporary times, AEC have become a prominent form of antenatal knowledge transmission in Aotearoa, Australia, Canada and the US [10]. Fabian et al. [12] attest that most health professionals recommend AEC to most expectant parents using the service. Indigenous Peoples, however, have lower rates of attendance at AEC [44]. Gagnon and Sandall [13] note that AEC have replaced previous Indigenous forms of knowledge transmission. “The existence of structured education in preparation for childbirth and parenthood has come about as traditional methods of information sharing have declined” (p.3).

Nolan and Hicks [11] proclaim AEC aims to create a “cohesive network amongst class members to enable them to support each other through the transition to parenthood. In this way, classes attempt to recreate the support which women traditionally found within extended families and local communities” (p. 186). AEC are firmly grounded in a social model of support for parents during pregnancy and the postnatal period [11]. Social support, assessment, and education are core programme components of Centering Pregnancy, an example of an AEC [45].

AEC are a significant cost to maternity services requiring careful evaluation [9]. Recently the effectiveness of AEC has come into question, with mixed findings on whether AEC have any impact on labour and birthing outcomes [9, 12, 46, 47] or effect on obstetric and psycho-social outcomes [10]. Nolan and Hicks [11] have stressed that antenatal education’s survival is “dependant upon its being perceived and evaluated as a broad educational intervention and not as an obstetric one. Its effectiveness needs to be audited according to educational criteria and not clinical” (p. 187). Gagnon and Sandall’s [13] review of studies demonstrated there is a wide spectrum of antenatal education, including variants on what education or information is delivered, how it is delivered, and to whom it is delivered.

AEC can vary in delivery mode, ranging from large lecture style classes, small classes, internet-based programmes, or one on one sessions [13]. The information taught also varies and can include topics about pregnancy, labour, birth, and parenting. Buultjens et al. [48] highlights the limited research investigating perceptions of the educational content currently communicated in antenatal service provision, resulting in inconsistency of AEC delivery.

Gagnon and Sandall [13] found AEC on offer typically attracted attendees who were “well educated women in the middle-to-upper socio-economic strata” (p. 4). Deeb-sossa and Kane’s [49] analysis of prenatal classes found “the content and messaging of these classes appears to have contributed to a societal tendency to make pregnant women, especially poor women and women of color, invisible…” (p. 380). Fabian et al. [12] suggest future research “should focus on current forms of antenatal education, with special focus on women of low socioeconomic status” (p.436).

AEC are considered an important opportunity to support positive antenatal health outcomes [11, 50, 51]. Maternal and infant health outcomes are statistically worse for Indigenous Peoples in Aotearoa, Australia, Canada and the US. Given the potential of Indigenous frameworks to enhance health and wellbeing to address antenatal inequities, and the legal and moral obligations to uphold Indigenous Peoples health sovereignty within these four countries, the expectation that Indigenous frameworks would at a minimum, be present in AEC classes is warranted. The colonial history of these Indigenous populations influences inequities of outcomes and drives how health systems are designed and delivered. AEC have replaced a traditional maternity system and have remained the dominant form of antenatal education transmission post colonisation, though delivery mode and content varies. Therefore, this paper provides the results of a systematic literature review to determine what extent Indigenous Peoples are prioritised and involved in antenatal education classes in Aotearoa, Australia, Canada, and US. Further, to understand the experience of Indigenous Peoples from these countries in antenatal education classes.

## Methods

Our team employed a qualitative meta-synthesis method to undertake this systematic review using a socio-critical lens. This socio-critical perspective acknowledges the environmental, social, cultural determinants of health, critique and social justice [52] and aligns to studies involving respondents’ perspectives and broader experiences of healthcare [53]. Alongside our approach we conducted a systematic review following the PRISMA 2009 guidelines.

### Search strategy

We used the PRISMA protocol to search the following databases: EBSCOhost, ProQuest Central, PubMed, PubMed Central (PMC), and Google Scholar search. For the purposes of this review, antenatal education classes (AEC) are defined as, ‘an organised and structured intervention delivering pregnancy related information and education on different health topics/areas’. Across and within countries, AEC can have interchangeable names therefore the following search terms were used: “antenatal education” OR “prenatal education” OR “antenatal classes” OR “prenatal classes” OR “birth preparation” OR “childbirth classes” *n* = 3291. We then added the following search terms; AND Indigenous *n* = 114; AND Australia *n* = 564; AND New Zealand *n* = 157; AND United States *n* = 950; AND Canada; AND ‘Maori’ OR ‘Aboriginal’ OR ‘First Nations’ OR ‘Native’ *n* = 229.

### Inclusion and exclusion criteria

Inclusion criteria were: 1) qualitative or mixed-methods studies based in Aotearoa New Zealand, Australia, Canada or the United States; 2) a primary focus or objective on an antenatal education class/intervention with a holistic focus; and 3) participants were end-users of intervention.

Exclusion criteria were: 1) non-English language studies; 2) published outside of the selection period between January 2008 and December 2018; and 3) studies that were resources for health professionals such as childbirth educators or nurses.

### Study selection

Title and abstracts of records identified from database and individual journal searches were screened, and articles not meeting the eligibility criteria were excluded. The full text of potentially eligible papers was reviewed, and only those meeting the eligibility criteria were included in the review.

### Risk of bias in individual studies

The methods, data quality, study context and other risks of bias in each eligible paper were assessed to ascertain their validity. Papers at risk of bias were identified, and their potential impact on the results was assessed.

Two publications were identified as being the same study but published in two different journals with subtle changes. The authors agreed that though they were similar they were included in the final selection and that if there was any impact on results this would be identified in the Results section of this article.

### Analysis

We applied a socio-critical lens to the qualitative meta-synthesis. Our team undertook an independent analytical process lead by the first author; followed by robust collaborative discussions with remaining authors. From these discussions we were able to identify key themes relevant to our research questions.

## Results

**Fig. 1:**
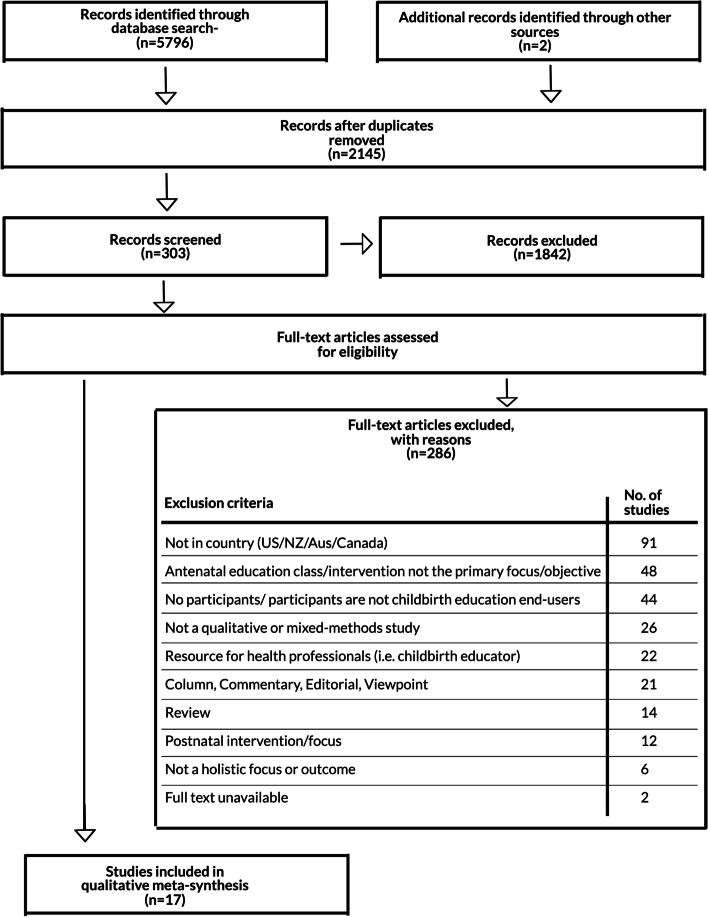
PRISMA flow chart study inclusion

The searches identified 5796 records in the EBSCOhost, ProQuest Central, PubMed and PubMed Central (PMC) databases, with a further two identified from Google Scholar (Fig. 1). After removing the duplicates there were 2145 initial records. A screening of each record was undertaken of the title and abstract with 1842 records excluded and 303 articles identified as potentially eligible for inclusion. The 303 full-text articles were then assessed for eligibility and 286 studies were excluded with reasons (Fig. 1). This yielded 17 papers considered eligible for inclusion in this review.

Table 1 is a summary of the 17 studies included in this review. In relation to our first review question, determine the extent of Indigenous Peoples prioritisation and involvement in antenatal education classes, we present first the results of how many studies recorded ethnicity data, and then identify which of those studies included Indigenous Peoples as participants. Of the reviewed manuscripts only nine of the 17 studies fully identified participants’ ethnicity. Two studies partially identified participants’ ethnicity describing their participants as “mostly Anglo-Australian” [54, 55]. The remaining six studies had no mention of participant ethnicity. Five studies had no involvement of Indigenous participants and it is unclear whether Indigenous participants were involved in 10 of the 17 studies. Consequently, only two studies definitively included Indigenous participants with both studies based in the United States.

**Table 1:** Summary of studies included in qualitative meta-synthesis

Author/s	Aim	Intervention/s	Research Approach		Participants			Country	Findings
			Methodology/Theoretical perspective	Methods	No.	Ethnicity of participants identified?	Indigenous Peoples included?		
Ateah [56]	Determine first-time expectant parents’ perceptions of a parent education intervention, their education needs, and preferred sources and modes of such education.	1 hr. in-person session carried out during the last class of a public health prenatal education series.	Quantitative study with some open-ended questions	Questionnaire	*n* = 16 women *n* = 15 men	Unknown	Unknown	United States	Most participants in study found content useful, planned to use it in caring for their infant, and indicated this information should be shared with all expectant mothers.
Auger et al. [57]	Examine the effectiveness of a participatory prenatal education program for low-income Latinas.	Group intervention with three components, 1) use of photonovels, 2) participatory education, and 3) lay educator model.	Community-based participatory research approach	Surveys, focus groups, medical records	*n* = 43	Fully (Latinas)	No	United States	Participants showed a significant increase in knowledge and confidence and reported an increase in social support, a deeper understanding of information, greater engagement, and behaviour change.
Bourget et al. [58]	The development and evaluation of an educational intervention that aimed to promote the development of a sense of mastery of anticipated of the anticipated paternal role in soon-to-be fathers.	4 educational sessions delivered to expectant fathers attending prenatal classes.	Preventive role supplementation conceptual framework	Questionnaire	*n* = 6	Unknown	Unknown	Canada	Participants highly appreciated the content and format of the educational intervention, developing a sense of mastery of the anticipated paternal role.
Broussard and Broussard [59]	Evaluate the Resource Center for Young Parents-To-Be project.	Six lessons for resource center young parents-to-be project.	Qualitative evaluation	Questionnaire	Unknown	Unknown	Unknown	United States	The adolescents who have attended have given the resource center and the nursing students high marks.
Fisher et al. [60]	Experiences of expectant mothers and their birth support partners participation in Mindfulness-based Child Birth education.	Mindfulness-based Child Birth education class.	Pedagogical approach	Focus group	*n* = 12 women *n* = 7 birth support partners	Unknown	Unknown	Australia	A sense of both ‘empowerment’ and ‘community’ were the essences of the experiences of MBCE for all participants.
Fitzgerald et al. [61]	Seek a better understanding of needs and access issues among pregnant low-income Hispanic women who attended a community prenatal education program.	Community prenatal education program.	Phenomenological inquiry	Focus groups	*n* = 8	Fully (Hispanic)	No	United States	Adequate and culturally appropriate health care services to pregnant Hispanic women in the greater Louisville metropolitan area is still lacking.
Gambrel and Piercy [62]	Understand the lived experiences of participants in the Mindful Transition to Parenthood Program.	4-week Mindful Transition to Parenthood Program.	Phenomenological inquiry	Semi-structured interviews	*n* = 26 (13x couples)	Fully (Caucasian and non-Hispanic, American Indian and Caucasian, Latino, Asian American, other, did not answer)	Yes	United States	Participants stated that the programme increased their acceptance and awareness, deeper connections with their partners, and led them to be more confident about becoming parents.
Gentles et al. [63]	Evaluate the TAPUAKI programme for the effectiveness and delivery of its curriculum to pregnant mothers.	TAPUAKI pregnancy and parenting programme.	Pacific talanoa methodological design. Qualitative thematic analysis .	Survey questionnaires, focus group interviews	*n* = 13	Fully (Samoan, Tongan, Cook Islands Māori)	No	New Zealand	Participants reported that their knowledge about pregnancy and parenting had increased as a result of the programme, with women responding positively and feeling a benefit through attending.
Koehn [64]	Describe and understand contemporary childbearing women’s perceptions of the role of childbirth education in preparing for birth.	Childbirth preparation class.	Grounded theory	Open-ended interviews	*n* = 9	Fully (Caucasian)	No	United States	Participant’s narratives support a relationship between childbirth education and readiness for the childbirth experience.
Levett et al. [65]	Gain insight into the experiences of women, partners and midwives who participated in the Complementary Therapies for Labour and Birth Study, an evidence based complementary medicine (CM) antenatal education course.	Complementary medicine antenatal education course.	Qualitative Study	In-depth interviews, focus group	*n* = 13	Fully (Caucasian, Asian)	No	Australia	Women used information about normal birth physiology from the course to make sense of labour, and to utilise the CM techniques to support normal birth and reduce interventions in labour. Women’s, partners’ and midwives’ experience of the course and its use during birth gave rise to supporting themes such as: working for normal; having a toolkit; and finding what works.
Liu et al. [66]	Examine how the CenteringPregnancy, a model of group prenatal care and childbirth education, influenced the birth experience of immigrant and minority women.	CenteringPregnancy group prenatal care and childbirth education.	Thematic analysis	In-depth interviews, surveys	*n* = 34	Fully (American Indian/Alaska Native, African American, Latina, White, Multi-racial)	Yes	United States	Participation in CenteringPregnancy model successfully equipped participants with a variety of pain and copping methods. Women reported high levels of satisfaction with their birth experiences.
Mackert et al. [67]	Explore the use of an e-health application to educate men about pregnancy-related health.	E-health application.	Qualitative study	Semi-structured interviews	*n* = 23	Fully (White, Hispanic, Asian, multiracial or other, Black)	Unknown	United States	The overwhelming positive reactions of participants both to the health issue, content, and design of the intervention is promising.
McNeil et al. [68]	Understand the central meaning of the experience of group prenatal care for women who participated in CenteringPregnancy; a forum for women to experience medical care and child birth education simultaneously and in a group setting.	Group prenatal care: forum for women to experience medical care and child birth education simultaneously.	Phenomenological approach	One-on-one interviews and/or group sessions	*n* = 12	Fully (Non-Caucasian)	Unknown	Canada	The central meaning of the experience of group prenatal care for women in this study was getting more than they realized they needed, with women gaining more from group prenatal care than from individual care.
Munro et al. [69]	Explore women’s preferences for a prenatal education program by text messaging.	SmartMom mHealth program for prenatal education.	Formative qualitative evaluation	Questionnaire, focus groups	*n* = 40	Unknown	Unknown	Canada	Participants perceived SmartMom to be highly acceptable and relevant for childbearing Canadian women.
Nash [54]	To examine how first-time fathers in rural Tasmania experienced father-only antenatal support/education groups.	Two classes, 1) government health service not-for-profit and 2) private company men’s antenatal education classes in a pub.	Masculinity	Semi-structured interviews	*n* = 25	Partially (Most Anglo-Australian)	Unknown	Australia	Father-only groups can be improved by accounting for multiple and complex constructions of masculinity, increasing the number of sessions offered and altering the structure to suit the audience.
Nash [55]	Explore how a cohort of 25 first-time fathers experienced 2 different father only antenatal support/education groups.	Two father-only antenatal support/education groups; 1) GBADC AND 2) Bubs and Pubs.	Masculinity	Semi-structured interviews	*n* = 25	Partially (Most Anglo-Australian)	Unknown	Australia	Antenatal education-support programs in Tasmanian fail to recognize the multiple, complex constructions of masculinity that characterize the current generation of expectant fathers. Father only programs can be improved by increasing the number of sessions offered and by altering the structure.
Spicer [70]	Interpret and understand how antenatal education, both with and without hypnosis, impacted a mother’s birthing experience.	Two classes 1) Traditional antenatal education and antenatal education with the inclusion of hypnosis.	Hermeneutic phenomenological and interpretive approach.	Interview	*n* = 12	Unknown	Unknown	Australia	Antenatal education affects the ability of both mother and partner to manage labour and childbirth to their perceived level of satisfaction. The inclusion of hypnosis in antenatal education provided mothers with a powerful and useful intervention.

Of the two studies where Indigenous Peoples were participants of AEC, American Indian made up 12% (*n* = 3) of total participants in one study [62]; whilst American Indian/Alaska Native made up 9% (*n* = 3) of total participants in the second study [66]. Our second review question was to understand the experience of Indigenous Peoples from these countries in AEC. Within these two studies the sample size of Indigenous Peoples was less than a quarter of the participant numbers. As a result of poor engagement of Indigenous Peoples in these AEC it was not possible to understand their experience of AEC.

## Discussion

This systematic review reveals that AEC in Aotearoa, Australia, Canada and US do not prioritise the engagement of, or experiences of, Indigenous Peoples with only two studies identifying Indigenous Peoples as participants. Furthermore, most of these studies paid no attention to ethnicity data collection and whether Indigenous Peoples were involved. The two studies that did collect ethnicity data were far from representative of the antenatal health inequities of each of the Indigenous population groups.

This review highlighted a lack of thought and consideration afforded to Indigenous Peoples of these countries. Indigenous Peoples’ health perspectives, sovereignty, and self-determination, all of which are fundamental rights of Indigenous Peoples [71], were not evident in the reviewed papers. This lack of consideration meant there was an absence of data pertaining to our second review question, which was to understand the experience of Indigenous Peoples in AEC. In so saying, the absence of this data has identified important areas that need to be addressed in order to improve antenatal health inequities.

### AEC need to collect quality ethnicity data

Identity is fundamental to Indigenous Peoples. Self-identification is the right of all ethic-cultural groups, as Chiriboga [72] explains “…to be recognized as different; to maintain their characteristic culture and their cultural patrimony, both tangible and intangible; and not be forced to belong to a different culture or to be unwillingly assimilated by it” (p.45). Article 33 section 1 of the United Nations Declaration on the Rights of Indigenous Peoples [73], states that “Indigenous peoples have the right to determine their own identity or membership in accordance with their customs and traditions” (p.10). Self-identification is the right to be counted [74] and asserting this right is shared by all Indigenous Peoples. This right was ignored in the AEC studies we canvassed. For Indigenous Peoples’ identity is central to good health and this oversight is a key contributor to maternal and infant health inequities.

Reporting of quality ethnicity data was not consistent across all studies, with eight of the 17 studies collecting no or partial ethnicity data. Quality ethnicity data collection is needed to monitor ethnic inequities in health and social outcomes [75]. If it is not completed accurately or even included at all, this will impede strategic implementation of health initiatives that aim to reduce avoidable deaths [76]. This process of ethnicity data collection is especially important for Indigenous Peoples of colonised countries where leading government/crown/state entities have legal and moral obligations to uphold their rights [75]. Without collecting ethnicity data there is no way to monitor accountability of these entities nor whether health equity is achieved. Access to ethnicity data also permits sovereignty for Indigenous Peoples, to measure and monitor their own vital health statistics, to determine priorities and conduct strategic future planning.

### AEC classes to benefit Indigenous Peoples

The majority of studies included in this review yielded positive responses from participants in relation to the AEC or intervention, however data on Indigenous experiences was not present in the manuscripts. There were three studies that targeted a specific ethnic group of peoples, each employing elements of culture and language in a manner that resonated with the intended participants. Findings from these studies showed participants highly valued and appreciated the respective interventions. This supports Laverack’s [77] statement that people “want to participate [in health interventions] and will do so in large numbers if they are properly engaged and have a shared interest in the program” (p.3). This targeted approach received participant endorsement resulting in life changing knowledge and behaviours.

In contrast, a study conducted by Nguyen et al. [78] based in the United States on women from racial or ethnic minority and low socioeconomic backgrounds, concluded that “despite reporting higher levels of prenatal health education on a variety of health-related topics, disadvantaged women continue to experience disparities in adverse birth outcomes suggesting that education is insufficient in promoting positive behaviors and birth outcomes” (p. 157). The preceding quote validates the need for targeted health approaches for intended participants and that inadequate content and mode of delivery of AEC can increase maternal and infant health inequities.

Responsive AEC are needed for Indigenous Peoples to engage intended communities and achieve health equity. The urgency to prioritise Indigenous interventions is clear. “The inconsistent progress in the health and well-being of Indigenous populations over time, and relative to non-Indigenous populations, points to the need for further efforts to improve the social, economic, and physical health of Indigenous peoples” ([27] p.1). Context-specific and relational approaches that privilege local Indigenous knowledge are shown to be more responsive in achieving health equity. Fijal and Beagan’s [79] literature synthesis of Indigenous perspectives on health found, “Indigenous knowledge and ways of life, Indigenous cultures, and Indigenous identities were all identified in the literature as critical to health and well-being…” (p. 220).

This review, albeit relatively brief compared to the depth of knowledge needed to understand the complexities and targeted approaches for each of the four Indigenous population groups (and their subgroups), highlights that Indigenous approaches share elements of commonality, resonating with one another. Hilgendorf et al. [80] goes further to iterate, “recent perspectives on Indigenous health have recognized language, culture, and values as central to well-being and recovery from historical trauma” (p.824). As health professionals and researchers, if the aim is to move beyond the exchange of knowledge and instead elicit behaviour change and empowerment, an approach that affects participants’ identity, where a meaningful connection is made, is needed.

### Barriers for publishing community health initiatives for Indigenous Peoples

This review highlights a lack of Indigenous focused AEC, however we acknowledge there are Indigenous led interventions within these countries, specifically regarding the revitalisation of traditional birthing practices [40, 43, 81]. There are several barriers and factors that may account for why interventions are not present in the literature. For many Indigenous communities publishing in scholarly journals may not be a priority or desire, or they lack support to share their findings on a global stage. Academic literature, specifically submission of articles into prestigious journals, is predominantly an activity for researchers, with many communities and health organisations focused on delivery of services rather than dissemination of findings in academic journals.

Indigenous researchers must navigate the complexities of academic processes that can at times be in direct opposition of Indigenous obligations [37, 82]. Publication is a familiar and encouraged process for Western scholars yet the constraints of publishing research data in journals is a barrier for many Indigenous researchers [83]. Tierney et al. [83] further argue that in some instances, non-Western scholars have undertaken processes to “accommodate or assimilate to Western standards” (p.296). Indigenous scholars must overcome the peculiarities of Western academia, including having to continuously defend and validate Indigenous knowledge [84, 85] whilst navigating the obligations of being first and foremost, an Indigenous person. In relation to Indigenous birthing revitalisation, there are several Indigenous scholars actively working in this academic space such as Simmonds [86], Moewaka Barnes et al. [2], Gabel [87] from Aotearoa. Indigenous researchers can overcome these challenges however it is an added complexity that contributes to the dominated Western studies.

### Strengths and limitations

Acknowledged above, much work that Indigenous health professionals are conducting in their communities is not published in academic sources or may have been excluded due to the limitations of a systematic review. Our team have found sources that show there is knowledge and experience surrounding traditional Indigenous maternity systems that was absent from the published literature, confirming that a wider body of anecdotal and grey literature exists [88].

## Conclusion

Indigenous Peoples of Aotearoa, Australia, Canada and US are not prioritised in antenatal education research, with only two of 17 studies identifying Indigenous participants. Of these two studies, Indigenous Peoples made up less than one quarter of participants. As a result of poor engagement and low participation numbers of Indigenous Peoples in these antenatal education classes it was not possible to understand the experience of Indigenous Peoples from these countries.

The absence of Indigenous Peoples’ data highlights a lack of consideration from both the researchers and developers of antenatal education classes, and subsequently, the rights of Indigenous Peoples’ health and sovereignty. The fundamental right for Indigenous Peoples to self-identification was severely lacking with six of the 17 studies disregarding the right to self-identification. Neglecting this process negatively impacts Indigenous Peoples as Durie [89] exclaims, “a secure Māori cultural identity is central to good health” (p. 189). The absence of identity in health stems from a Western definition of health, which is at odds with Indigenous and holistic health perspectives. Collecting quality ethnicity data is an essential first step toward upholding the fundamental right of Indigenous Peoples’ to be counted.

Of the studies analysed in this review, a need for cultural embeddedness rather than as an add-on was demonstrated by three of the studies, each with interventions that targeted a specific ethnic population. Those studies embedded elements such as identity, language, and a feedback loop from participants for the intervention to be strengthened. A dedicated commitment where the intervention designers do not see themselves as ‘the expert’ but genuinely valuing the expertise and knowledge of their participants. These elements align to Indigenous health models and provide a basis for authentic health intervention design.

To address the stark inequities of Indigenous Peoples antenatal health and wellbeing statistics, there is a clear need for more studies driven by Indigenous Peoples attending to Indigenous ways of knowing. This is not to say that Indigenous interventions are not being delivered for these priority communities, but instead highlights the lack of support and little emphasis for Indigenous knowledge in scholarly sources. Targeted Indigenous interventions that consider culture, language, and wider aspects of holistic health provide a solution moving forward. These solutions must be privileged.

## Data Availability

Not applicable.

## References

[1] Villar J, Ba’aqeel H, Piaggio G, Lumbiganon P, Belizán JM, Farnot U (2001). WHO antenatal care randomised trial for the evaluation of a new model of routine antenatal care. Lancet.

[2] Moewaka Barnes H, Moewaka Barnes A, Baxter J, Crengle S, Pihama L, Ratima MM (2013). Hapū ora: wellbeing in the early stages of life.

[3] Barker DJ (2007). The origins of the developmental origins theory. J Intern Med.

[4] Barker DJ, Osmond C, Golding J, Kuh D, Wadsworth ME (1989). Growth in utero, blood pressure in childhood and adult life, and mortality from cardiovascular disease. Br Med J.

[5] Russ SA, Larson K, Tullis E, Halfon N (2014). A lifecourse approach to health development: implications for the maternal and child health research agenda. Matern Child Health J.

[6] Cliff D, Deery R (1997). Too much like school: social class, age, marital status and attendance/non-attendance at antenatal classes. Midwifery.

[7] Ahldén I, Ahlehagen S, Dahlgren L, Josefsson A (2012). Parents’ expectations about participating in antenatal parenthood education classes. J Perinat Educ.

[8] Detman LA, Quinn GP, Ellery J, Wallace K, Jeffers D (2008). Case study: consumer and provider perceptions of offered anticipatory guidance during prenatal care. J Commun Healthc.

[9] Ferguson S, Davis D, Browne J (2013). Does antenatal education affect labour and birth? A structured review of the literature. Women Birth.

[10] Brixval CS, Axelsen SF, Andersen SK, Due P, Koushede V (2014). The effect of antenatal education in small classes on obstetric and psycho-social outcomes: a systematic review and meta-analysis protocol. Syst Rev.

[11] Nolan ML, Hicks C (1997). Aims, processes and problems of antenatal education as identified by three groups of childbirth teachers. Midwifery.

[12] Fabian HM, Rådestad IJ, Waldenström U (2005). Childbirth and parenthood education classes in Sweden. Women’s opinion and possible outcomes. Acta Obstet Gynecol Scand.

[13] Gagnon AJ, Sandall J (2007). Individual or group antenatal education for childbirth or parenthood, or both. Cochrane Database Syst Rev..

[14] Durey A, Thompson SC (2012). Reducing the health disparities of indigenous Australians: time to change focus. BMC Health Serv Res.

[15] Lafontaine A (2018). Indigenous health disparities: a challenge and an opportunity. Can J Surg.

[16] Marrone S (2007). Understanding barriers to health care: a review of disparities in health care services among indigenous populations. Int J Circumpolar Health.

[17] Bramley D, Hebert P, Tuzzio L, Chassin M (2005). Disparities in indigenous health: a cross-country comparison between New Zealand and the United States. Am J Public Health.

[18] Blackwell CC, Moscovis SM, Gordon AE, All Madani OM, Hall ST, Gleeson M (2004). Ethnicity, infection and sudden infant death syndrome. FEMS Immunol Med Microbiol.

[19] Godoy M, Maher M (2022). A ten-year retrospective case review of risk factors associated with sleep-related infant deaths. Acta Paediatr.

[20] Mitchell E (2009). SIDS: past, present and future. Acta Paediatr.

[21] Walker K (2019). Issues of tobacco, alcohol and other substance abuse for Māori: report commissioned by the Waitangi Tribunal for stage 2 of the health services and outcomes kaupapa inquiry (Wai 2575).

[22] Filoche S, Garrett S, Stanley J, Rose S, Robson B, Elley CR (2013). Wāhine hauora: linking local hospital and national health information datasets to explore maternal risk factors and obstetric outcomes of New Zealand Māori and non-Māori women in relation to infant respiratory admissions and timely immunisations. BMC Pregnancy Childbirth.

[23] King M, Smith A, Gracey M (2009). Indigenous health part 2: the underlying causes of the health gap. Lancet.

[24] Greenwood M, De Leeuw S, Lindsay N (2018). Challenges in health equity for Indigenous peoples in Canada. Lancet.

[25] Hirai AH, Hayes DK, Taualii MM, Singh GK, Fuddy LJ (2013). Excess infant mortality among native Hawaiians: identifying determinants for preventive action. Am J Public Health.

[26] Dwayne M (2021). The Indigenous world 2021.

[27] Cooke M, Mitrou F, Lawrence D, Guimond E, Beavon D (2007). Indigenous well-being in four countries: an application of the UNDP’S Human Development Index to Indigenous Peoples in Australia, Canada, New Zealand, and the United States. BMC Int Health Hum Rights.

[28] Reid P, Robson B, Robson B, Harris R, Te Ropu Rangahau Hauora a Eru Pomare (2007). Understanding health inequities. Hauora: Màori standards of health IV. A study of the years 2000–2005.

[29] Boddington P, Räisänen U (2009). Theoretical and practical issues in the definition of health: insights from Aboriginal Australia. J Med Philos.

[30] Bourke S, Wright A, Guthrie J, Russell L, Dunbar T, Lovett R (2018). Evidence review of Indigenous culture for health and wellbeing. Int J Health Wellness Soc.

[31] Chakanyuka C, Bacsu J-DR, DesRoches A, Dame J, Carrier L, Symenuk P (2022). Indigenous-specific cultural safety within health and dementia care: a scoping review of reviews. Soc Sci Med.

[32] King A, Turia T (2002). He korowai Oranga: Maori health strategy.

[33] Reid JB, Taylor K (2011). Indigenous mind: a framework for culturally safe Indigenous health research and practice. Aborig Isl Health Work J.

[34] Koithan M, Farrell C (2010). Indigenous native American healing traditions. J Nurse Pract.

[35] Durie M, Elder H, Tapsell R, Lawrence M, Bennett S (2018). Maea te Toi Ora: Māori health transformations.

[36] Reid P, Cormack D, Paine S-J (2019). Colonial histories, racism and health—the experience of Māori and Indigenous peoples. Public Health.

[37] Smith LT (1999). Decolonizing methodologies: research and indigenous peoples.

[38] Brown H, Varcoe C, Calam B (2011). The birthing experiences of rural Aboriginal women in context: implications for nursing. Can J Nurs Res Arch..

[39] Adams K, Faulkhead S, Standfield R, Atkinson P (2018). Challenging the colonisation of birth: Koori women’s birthing knowledge and practice. Women Birth.

[40] Simmonds N, Gabel K, Hutchings J, Lee-Morgan J (2016). Ūkaipō: decolonisation and Māori maternities. Decolonization in Aotearoa: education, research and practice.

[41] Clarke A (2012). Born to a changing world: childbirth in nineteenth-century New Zealand.

[42] Ware F (2014). Whānau kōpepe: a culturally appropriate and family focused approach to support for young Māori (Indigenous) parents. J Indig Soc Dev..

[43] Simmonds N, Roberston M, Tsang P (2016). Transformative maternities: Indigenous stories as resistance and reclamation in Aotearoa New Zealand. Everyday knowledge, education and sustainable futures.

[44] Dwyer S (2009). Childbirth education: antenatal education and transitions of maternity care in New Zealand.

[45] Rising SS (1998). Centering pregnancy: an interdisciplinary model of empowerment. J Nurse Midwifery.

[46] Howharn C (2008). Effects of childbirth preparation classes on self-efficacy in coping with labor pain in Thai primiparas [PhD].

[47] Leach J, Bowles B, Jansen L, Gibson M (2017). Perceived benefits of childbirth education on future health-care decision making. J Perinat Educ.

[48] Buultjens M, Murphy G, Robinson P, Milgrom J, Monfries M (2017). Women’s experiences of, and attitudes to, maternity education across the perinatal period in Victoria, Australia: a mixed-methods approach. Women Birth.

[49] Deeb-sossa N, Kane H (2017). Pregnancy without women: lessons from childbirth classes. Sex Res Soc Policy.

[50] Nolan M (2012). Before we begin. The importance of antenatal education. Pract Midwife.

[51] Artieta-Pinedo I, Paz-Pascual C, Grandes G, Remiro-Fernandezdegamboa G, Odriozola-Hermosilla I, Bacigalupe A (2010). The benefits of antenatal education for the childbirth process in Spain. Nurs Res.

[52] Shelley K, McCuaig L (2020). Socio-critical lenses and threshold concepts in health, sport and physical education teacher education. Sport Educ Soc.

[53] Lachal J, Revah-Levy A, Orri M, Moro MR (2017). Metasynthesis: an original method to synthesize qualitative literature in psychiatry. Front Psychiatry.

[54] Nash M (2018). Addressing the needs of first-time fathers in Tasmania: a qualitative study of father-only antenatal groups. Aust J Rural Health.

[55] Nash M (2018). “It’s just good to get a bit of man-talk out in the open”: men’s experiences of father-only antenatal preparation classes in Tasmania, Australia. Psychol Men Masc.

[56] Ateah CA (2013). Prenatal parent education for first-time expectant parents:“making it through labor is just the beginning…”. J Pediatr Health Care.

[57] Auger SJ, Verbiest S, Spickard JV, Simán FM, Colindres M (2015). Participatory group prenatal education using photonovels: evaluation of a lay health educator model with low-income Latinas. J Particip Med.

[58] Bourget M, Héon M, Aita M, Michaud M (2017). An educational intervention to support the development of a sense of mastery of the anticipated paternal role in expectant fathers: a clinical project. J Perinat Educ.

[59] Broussard AB, Broussard BS (2009). Designing and implementing a parenting resource center for pregnant teens. J Perinat Educ.

[60] Fisher C, Hauck Y, Bayes S, Byme J (2012). Participant experiences of mindfulness-based childbirth education: a qualitative study. BMC Pregnancy Childbirth.

[61] Fitzgerald EM, Cronin SN, Boccella SH (2016). Anguish, yearning, and identity: toward a better understanding of the pregnant Hispanic woman’s prenatal care experience. J Transcult Nurs.

[62] Gambrel LE, Piercy FP (2015). Mindfulness-based relationship education for couples expecting their first child—part 2: phenomenological findings. J Marital Fam Ther.

[63] Gentles D, Fa’alili-Fidow J, Dunlop A, Roberts M, Ikihele A, Percival T (2016). Evaluation of the pilot TAPUAKI Pacific pregnancy and parenting education programme. Pac J Reprod Health.

[64] Koehn M (2008). Contemporary women’s perceptions of childbirth education. J Perinat Educ.

[65] Levett KM, Smith CA, Bensoussan A, Dahlen HG (2016). The complementary therapies for labour and birth study making sense of labour and birth – experiences of women, partners and midwives of a complementary medicine antenatal education course. Midwifery.

[66] Liu R, Chao MT, Jostad-laswell A, Duncan LG (2017). Does CenteringPregnancy group prenatal care affect the birth experience of underserved women? A mixed methods analysis. J Immigr Minor Health.

[67] Mackert M, Guadagno M, Lazard A, Donovan E, Rochlen A, Garcia A (2017). Engaging men in prenatal health promotion: a pilot evaluation of targeted e-health content. Am J Mens Health.

[68] McNeil DA, Vekved M, Dolan SM, Siever J, Horn S, Tough SC (2012). Getting more than they realized they needed: a qualitative study of women’s experience of group prenatal care. BMC Pregnancy Childbirth.

[69] Munro S, Hui A, Salmons V, Solomon C, Gemmell E, Torabi N (2017). SmartMom text messaging for prenatal education: a qualitative focus group study to explore Canadian women’s perceptions. JMIR Public Health Surveill.

[70] Spicer R (2014). My body, my birth, my baby: the experience of childbirth for first-time mothers who have undertaken traditional antenatal education and those who have included hypnosis. Aust J Clin Hypnother Hypn.

[71] Daes E-IA (2008). An overview of the history of indigenous peoples: self-determination and the United Nations. Camb Rev Int Aff.

[72] Chiriboga OR (2006). The right to cultural identity of indigenous peoples and national minorities: a look from the Inter-American System. Sur Revista Internacional de Direitos Humanos.

[73] United Nations General Assembley (2007). United Nations declaration on the rights of indigenous peoples.

[74] Robson B, Purdie G, Cram F, Simmonds S (2007). Age standardisation – an indigenous standard?. Emerg Themes Epidemiol.

[75] Yao ES, Meissel K, Bullen P, Atatoa-Carr P, Clark TC, Morton SM (2021). Classifying multiple ethnic identifications. Demogr Res.

[76] Freemantle J, Ring I, Arambula Solomon TG, Gachupin FC, Smylie J, Cutler TL (2015). Indigenous mortality (revealed): the invisible illuminated. Am J Public Health.

[77] Laverack G (2017). The challenge of behaviour change and health promotion. Challenges.

[78] Nguyen MN, Siahpush M, Grimm BL, Singh GK, Tibbits MK (2019). Women from racial or ethnic minority and low socioeconomic backgrounds receive more prenatal education: results from the 2012 to 2014 Pregnancy Risk Assessment Monitoring System. Birth.

[79] Fijal D, Beagan BL (2019). Indigenous perspectives on health: integration with a Canadian model of practice. Can J Occup Ther.

[80] Hilgendorf A, Anahkwet, Gauthier J, Krueger S, Beaumier K, Corn R (2019). Language, culture, and collectivism: uniting coalition partners and promoting holistic health in the Menominee Nation. Health Educ Behav.

[81] Simmonds N, Tait Neufeld H, Cidro J (2019). Honouring our ancestors: reclaiming the power of Māori maternities. Indigenous experiences of pregnancy and childbirth.

[82] Smith LT, Denzin NK, Lincoln YS (2005). On tricky ground: researching the native in an age of uncertainty. The Sage handbook of qualitative research.

[83] Tierney RJ, Smith GH, Kan W (2021). Global literacies research diversity: a manifesto for change. J Lit Res.

[84] Battiste M (2005). Indigenous knowledge: foundations for first nations. WINHEC International Journal of Indigenous Education Scholarship..

[85] Durie M (2004). Understanding health and illness: research at the interface between science and indigenous knowledge. Int J Epidemiol.

[86] Simmonds N (2009). Mana wahine geographies: spiritual, spatial and embodied understandings of Papatūānuku [Masters Thesis].

[87] Gabel K (2013). Poipoia te tamaiti ki te ūkaipō [PhD].

[88] Graham R, Masters-Awatere B (2020). Experiences of Māori of Aotearoa New Zealand’s public health system: a systematic review of two decades of published qualitative research. Aust N Z J Public Health.

[89] Durie M (1998). Whaiora. Māori health development.

